# Supplementary material for: Small molecule splicing modifiers with systemic HTT-lowering activity
Source: Nat Commun. 2021 Dec 15;12:7299. doi: 10.1038/s41467-021-27157-z (PMC8674292; doi:10.1038/s41467-021-27157-z)
Supplement: Supplementary file 1 — Supplementary Information [file 41467_2021_27157_MOESM1_ESM.pdf]

Supplementary Information for “Small molecule splicing modifiers with systemic HTT-lowering activity”

a

Compound	Structure	HTT protein lowering activity (IC_{50}) μM	Cytotoxicity (CC_{50}) μM	Mechanism
HTT-A	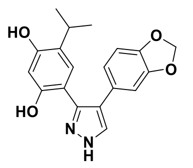	1.4	12	HSP90 inhibitor
HTT-B	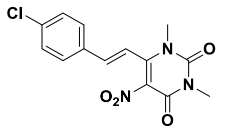	2.8	15	Unknown

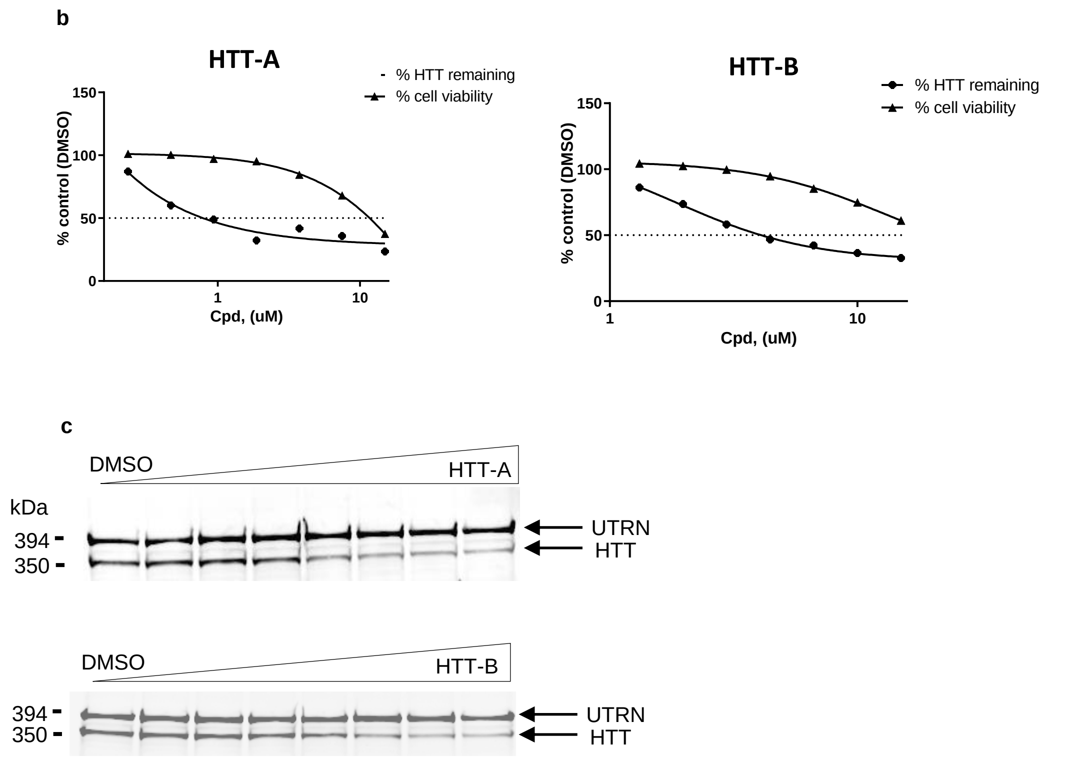

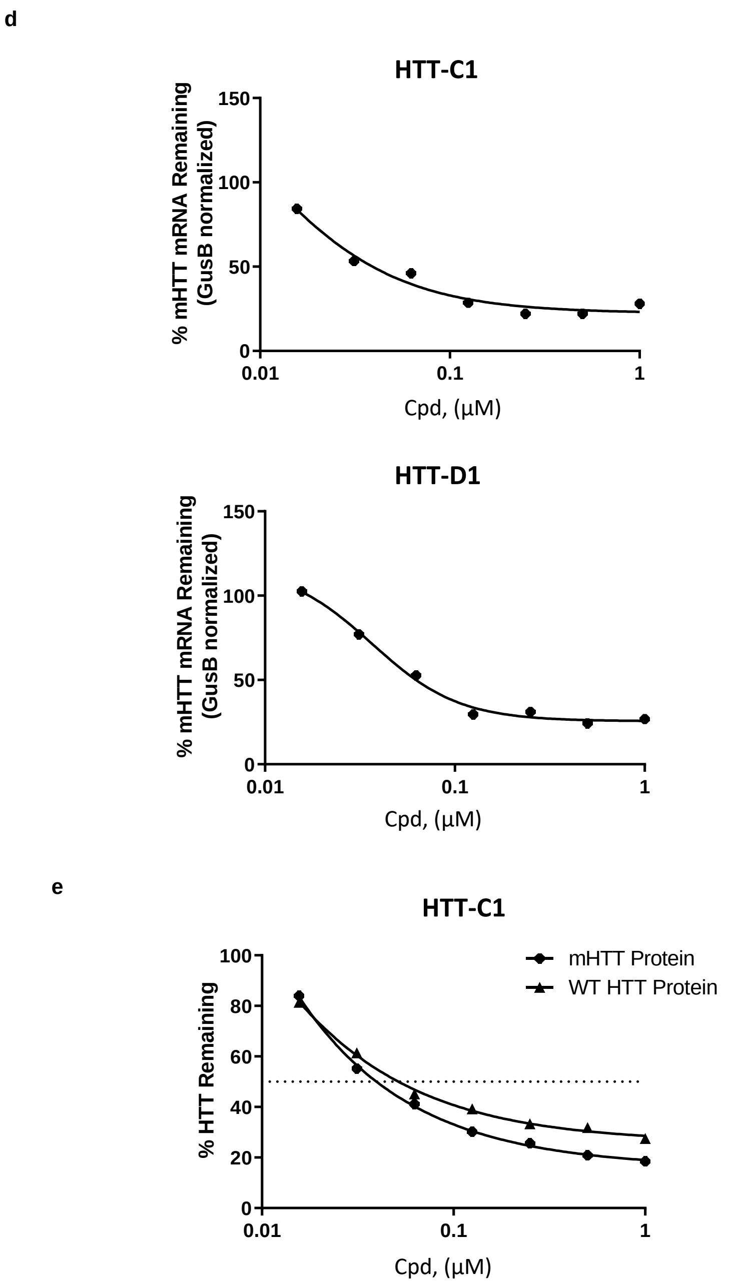

Supplementary fig. 1. Compounds identified with huntingtin (HTT)-lowering activity

a Chemical structure of HTT-A and HTT-B. **b** Electrochemiluminescence (ECL) analysis of total HTT protein from fibroblasts isolated from a homozygous patient with Huntington’s disease (HD) (GM04857) after 96 hrs of continuous treatment with HTT-A and HTT-B (0.01–10.0 μM). Representative graphs show percent HTT remaining relative to the dimethyl sulphoxide (DMSO) control. Cell-viability assays were performed in parallel. Data represent mean of two (n=2) biologically independent samples per data point. **c** Western blot of HTT protein in patient fibroblasts after treatment with HTT-A and HTT-B. Utrophin (UTRN) was used as a loading control. The western blot data was with a representative compound from each class (tested at multiple concentrations) from a single experiment. **d** Reverse transcription-quantitative polymerase chain reaction (RT-qPCR) analysis of *HTT* mRNA in patient fibroblasts after 24 hrs of treatment with HTT-C1 and HTT-D1 (0.01–1.0 μM). Representative graphs show percent *HTT* mRNA remaining normalised to beta-glucuronidase (*GUSB)* mRNA levels. Data represent mean of two (n=2) biologically independent samples per data point. **e** ECL analysis of wild-type (wt) and mutant HTT protein from control fibroblasts after treatment with HTT-C1 (0.01–1.0 μM). Data represent mean of two (n=2) biologically independent samples per data point.

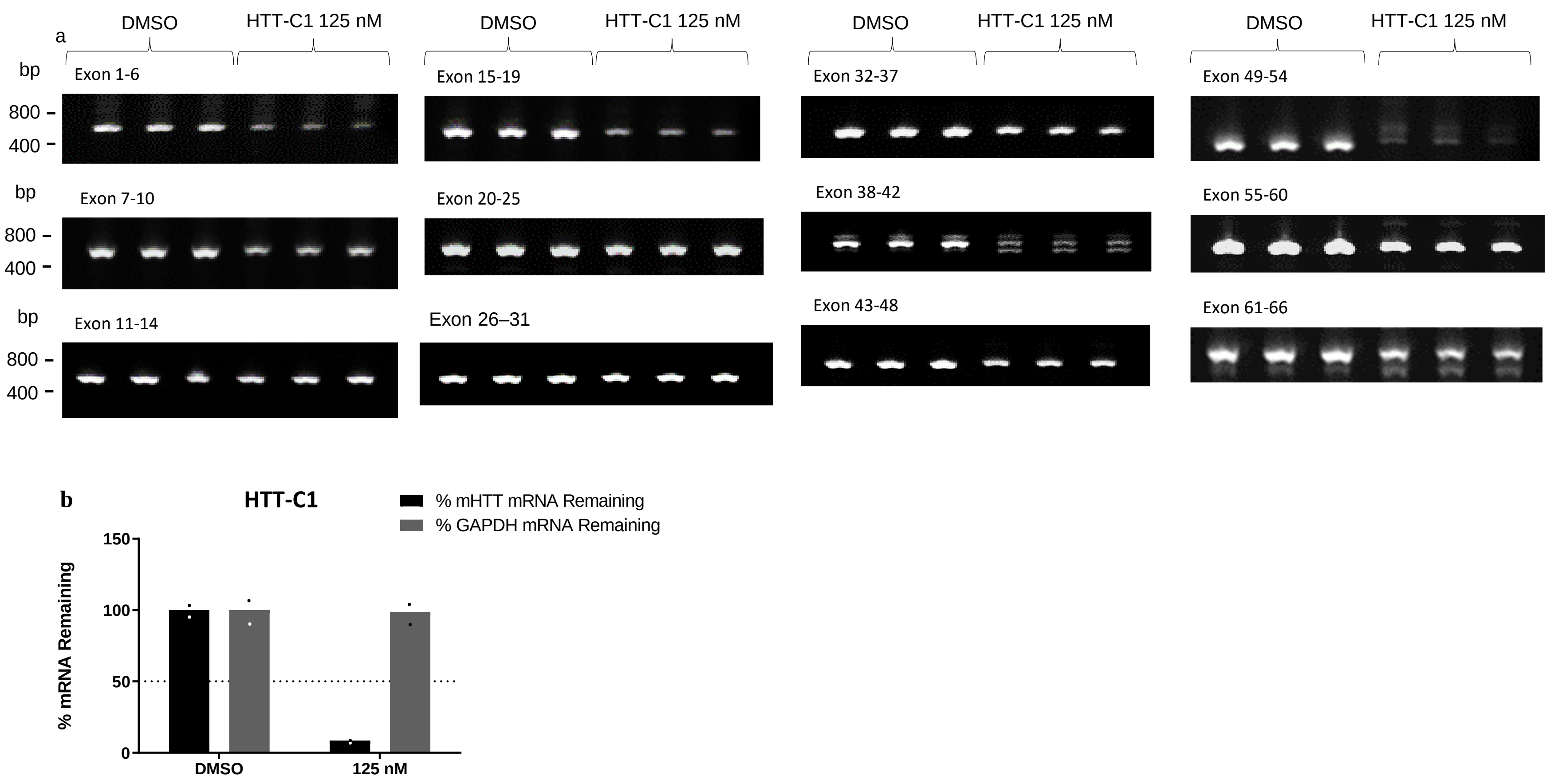

c

Intron	Read counts						JEI						ΔJEI (Compound vs. DMSO)	*P* value
	DMSO			Compound			DMSO			Compound				
**1**	3,242	4,972	2,525	2,550	3,451	3,534	100	100	100	100	100	99.92	-0.03	0.37
**2**	8,285	10,207	6,558	14,890	19,904	16,230	99.88	99.88	99.88	99.62	99.65	99.86	-0.17	0.09
**…**														
**49**	5,264	8,037	4,115	1,244	1,472	1,385	100	99.95	100	68.58	75.53	78.03	-25.94	0.00078
**…**														
**65**	4,236	3,766	3,193	1,358	1,163	1,804	99.98	100	100	99.93	99.74	99.94	-0.12	0.13
**66**	5,404	6,486	3,848	1,900	1,763	1,961	100	99.95	100	100	100	100	0.02	0.37

Supplementary fig. 2. Effect of compounds on splicing of the huntingtin *(HTT)* mRNA

a Reverse transcription-polymerase chain reaction (RT-PCR) analysis of *HTT* mRNA after 24-hr treatment with 125 nM HTT-C1 or dimethyl sulphoxide (DMSO) in patient derived B-lymphocytes (GM04856 cells). **b** Reverse transcription-quantitative polymerase chain reaction (RT-qPCR) analysis of *HTT* mRNA in B-lymphocytes from the same patient (GM04856 cells) after 24 hrs of treatment with DMSO control or HTT-C1 (125 nM). Representative graphs show percent HTT remaining relative to DMSO control; normalised to housekeeping gene, glyceraldehyde-3-phosphate dehydrogenase *(GAPDH)*. Data represent mean of two (n=2) biologically independent samples per data point. **c** Junction expression index (JEI) of intron 49 and a selection of other introns. *P*-value is based on a two-sided Student's t-test without adjustments for multiple comparisons. ΔJEI is the difference of average JEI for compound group (n=3) and DMSO group (n=3).

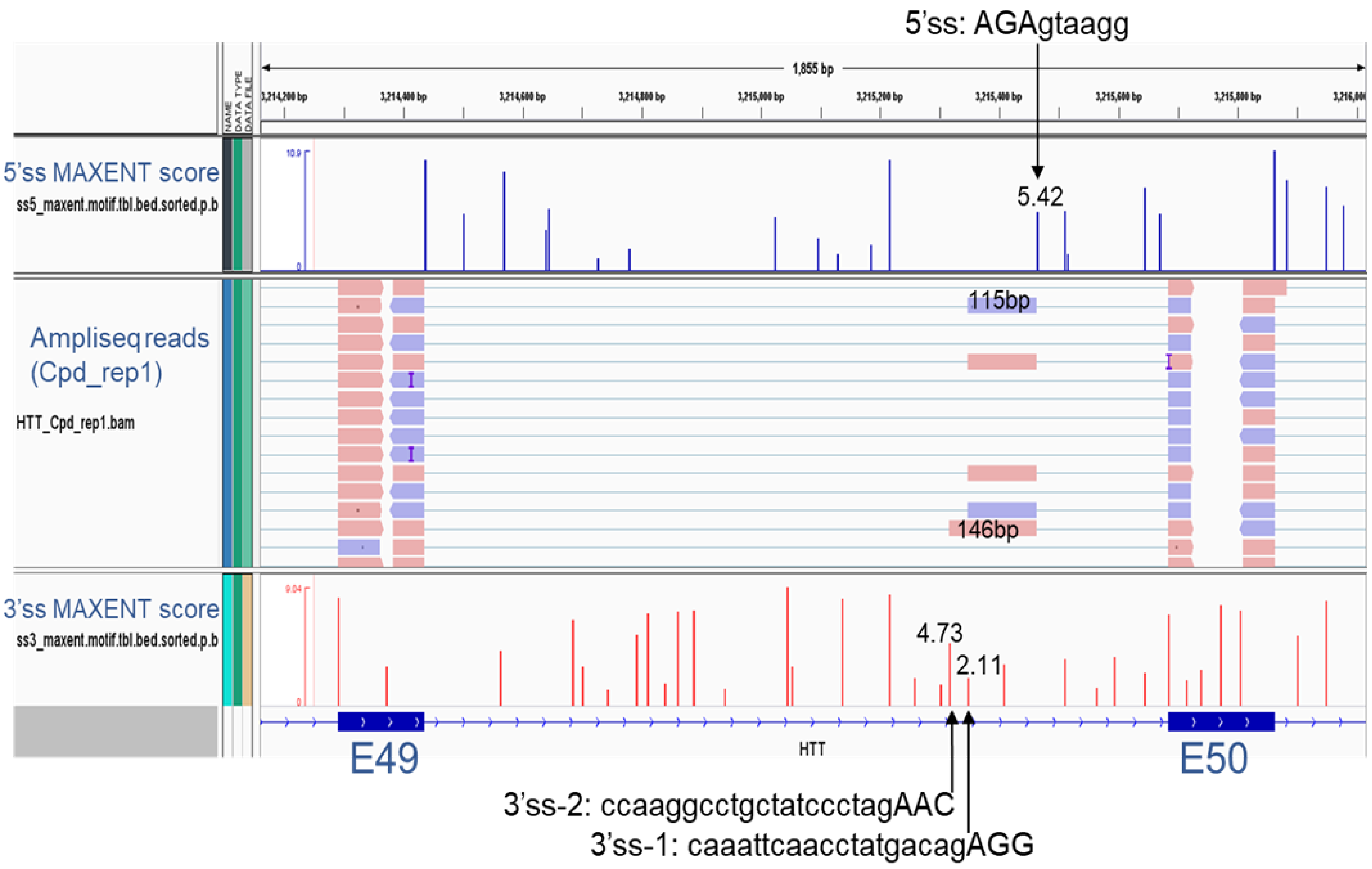

Supplementary fig. 3. Analysis of the human huntingtin gene *(HTT)* psiExon 49 and compound-induced inclusion of premature stop-codon leading to nonsense-mediated mRNA decay

Integrative Genomics Viewer visualisation of AmpliSeq read mapping of one of the HTT-C1-treated cells. The inclusion of psiExon49a can be seen by a few reads in the view. The 5' splice sites (ss) and 3'ss sequences and their MAXENT scores are also shown. The figure also indicated the reads supporting two different sizes of psiExon (115nt and 146nt) comprised of the using the same 5'ss, but different 3'ss 31 nucleotides apart from each other.

a

Sample	Raw read #
DMSO.1	30,837,054
DMSO.2	33,824,807
DMSO.3	33,152,129
DMSO.4	39,314,715
HTT-C2.100 nM.1	40,710,854
HTT-C2.100 nM.2	41,729,312
HTT-C2.24 nM.1	39,741,355
HTT-C2.24 nM.2	40,477,860

b

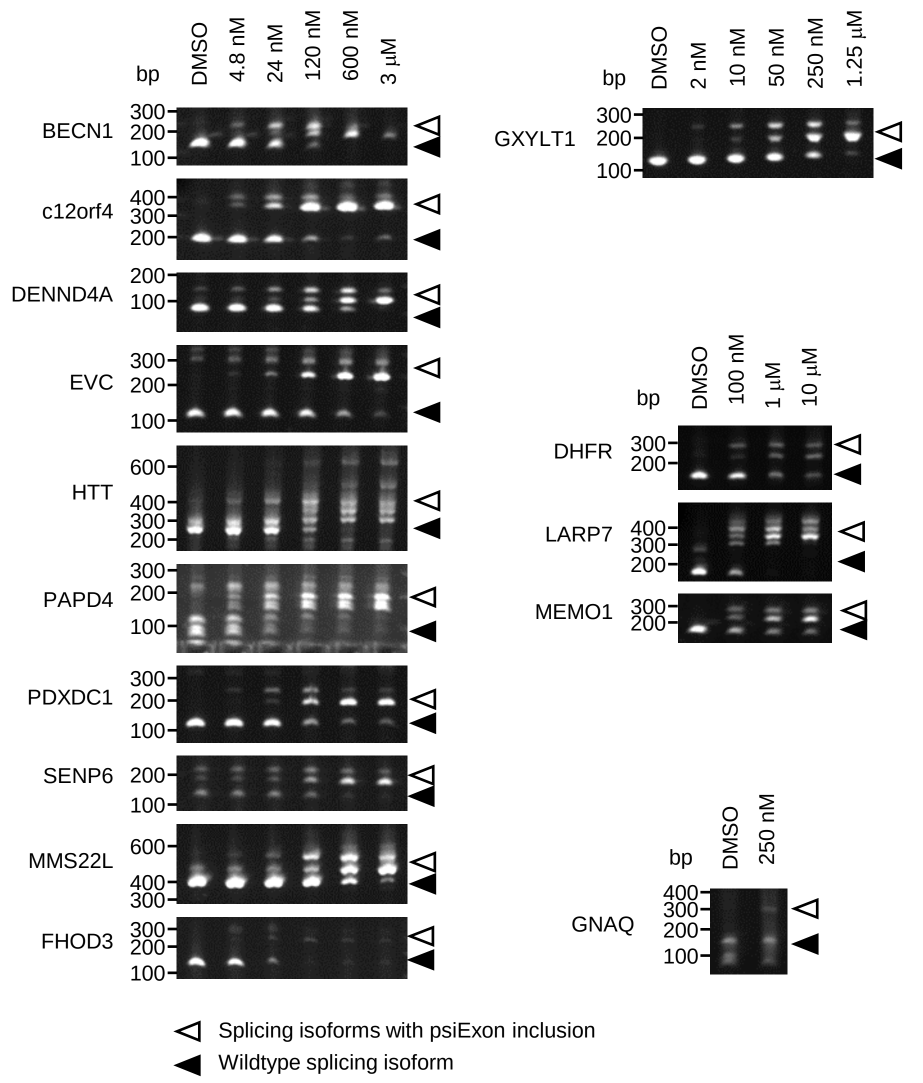

Supplementary fig. 4. RNA-Seq analysis of transcriptome changes

a RNA-Seq analysis of transcriptome changes in human SH-SY5Y cells treated with a close analogue of HTT-C1, HTT-C2 or control dimethyl sulphoxide (DMSO). Raw read # shown for each treatment. **b** Reverse transcription-polymerase chain reaction (RT-PCR) results showing validation of RNA-Seq-identified psiExon inclusion events in the human huntingtin gene (*HTT)* and several other genes following treatment with HTT-C2 or control (DMSO). The data is from multiple dose-response experiments; most primer sets have been used multiple times (n>3) on independent biological replicates.

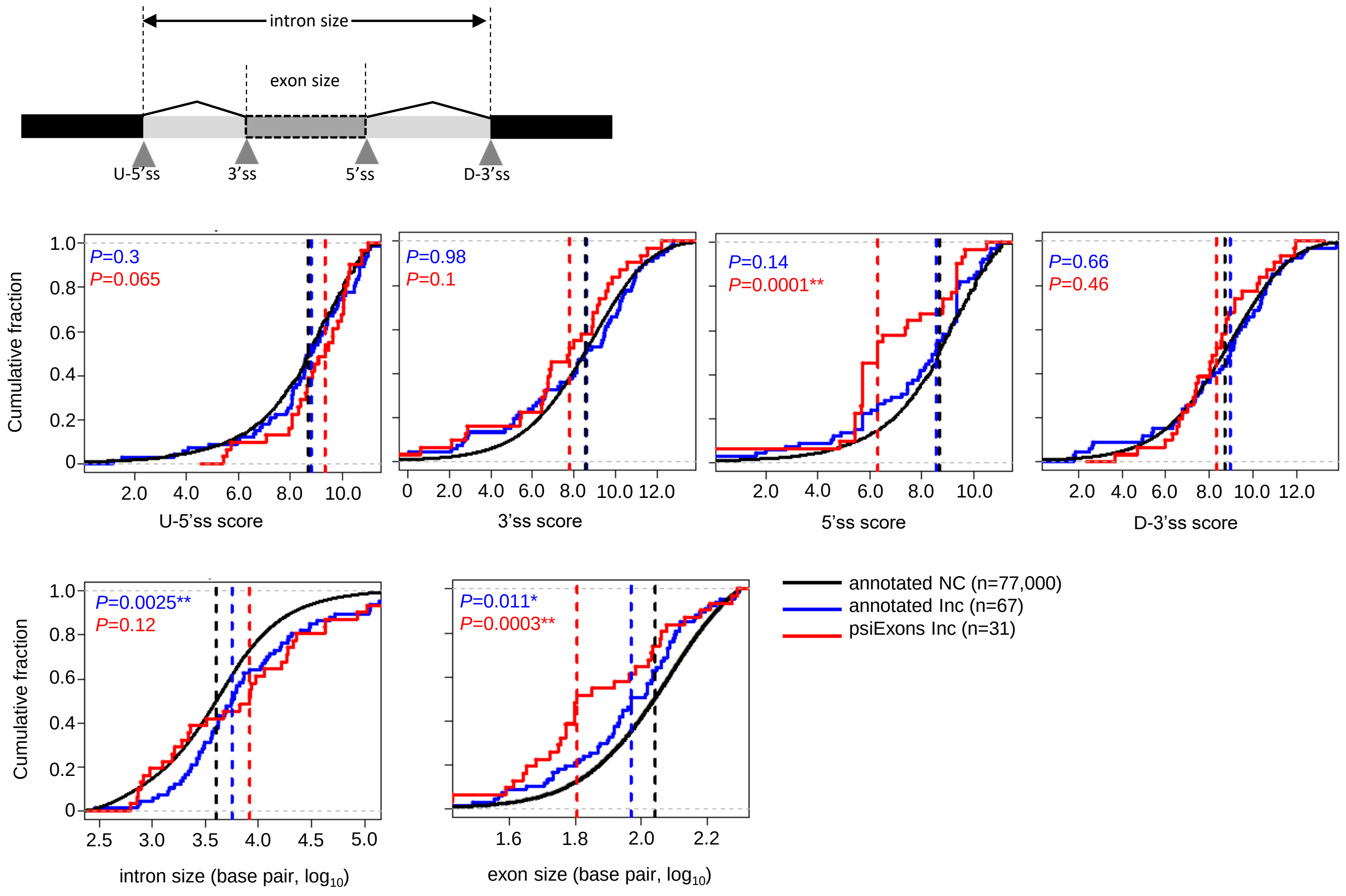

Supplementary fig. 5. Features of psiExons activated by HTT-C2

Analysis of 31 novel psiExon inclusion events identified through RNA-Seq analysis, showing that these psiExons are shorter and have significantly weaker 5'splice sites (ss) than annotated unaffected exons. *P*-values are based on Wilcoxon rank-sum test comparing a group of exons with "annotated NC" group exons. *P*-values are not adjusted for multiple comparisons. The median value of each group was shown as a dotted vertical line.

Compound	Structure	SMN protein increase ($EC_{1.5X}$), nM*	HTT protein lowering activity (IC_{50}), nM
HTT-C2	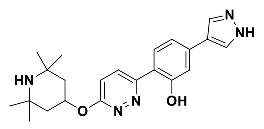	47	12
SMN-C3	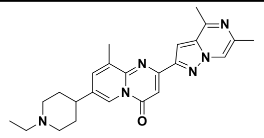	89	2043

Supplementary fig. 6. Comparing survival motor neuron (SMN) and huntingtin (HTT) activities for HTT-C2 and SMN-C3

The methods and conditions utilized to perform the homogeneous time resolved fluorescence survival motor neuron (HTRF SMN) protein assay in patient fibroblasts GM03813 (Coriell Institute) were described previously (See Naryshkin *et al*, 2014[1])

a

Sample	Raw read #
mock.1	27,953,629
mock.2	40,095,473
U1-GA.1	38,286,691
U1-GA.2	38,783,671

b

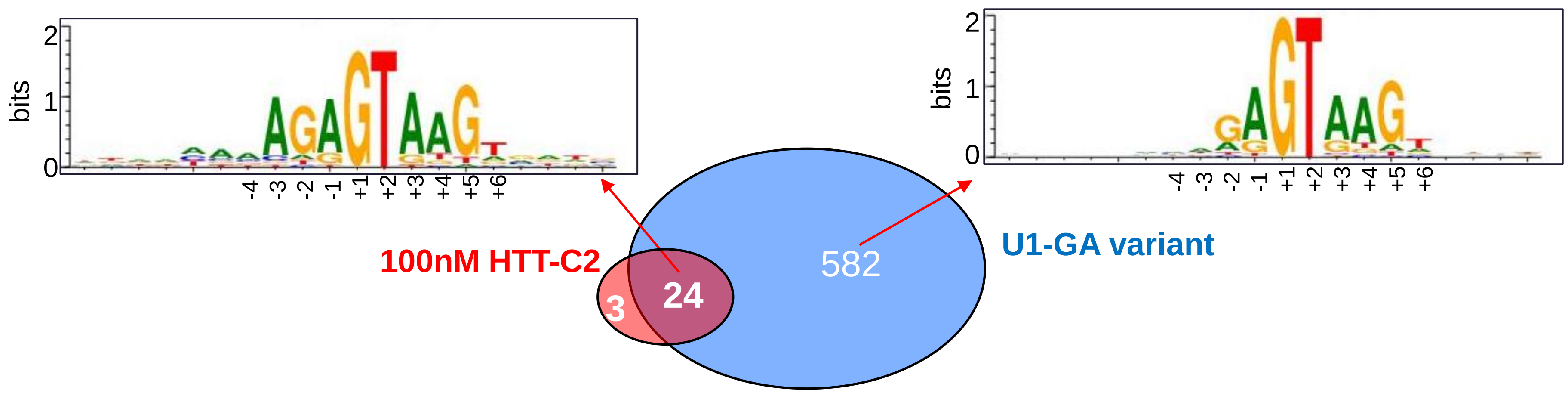

Supplementary fig. 7. psiExons identified from U1-GA variant treatment of HEK293 cells

a Read raw counts of the RNA-Seq from U1-GA variant treatment or mock controls. **b** Venn diagram showing the overlap of psiExons identified from 100nM HTT-C2 treatment and U1-GA variant treatment. Sequence logo of the 5' splice sites (ss) sequences were also shown for indicated subsets of psiExons.

a Multiple species sequence alignment of *HTT*-psiExon49a 5' and 3' splice sites

3'ss (hs -50 to +12):

```
caaggcctgctatccctagaacccacgctctCAAATTCAAC--------C--------------------------------TATGACAG(AGGCAAGCCCTG Human
caaggcctgctatccctagaacccacgctctCAAATTCAAC--------C--------------------------------TATGACAG(AGGCAAGCCCTG Chimp
caaggcctgctatccctagaacccacgctctCAAATTCAACCTCTGAGGC--------------------------------TATGACAG(AGGCAAGCCCCG Gorilla
caaggcctgctatccctagaacccatgctctCAAATTCAACCTCTGAGGC--------------------------------TATGACAG(A-GCAGGCCCCG Orangutan
caaggcctgctatccctagaacccacgctctCAAATTCAACCTCTGAGGC--------------------------------TATGACAG(AGGCAAGCCCCA Gibbon
caaggcctgctatccctagaacccacgctctCAAATTCAACCTCTGAGGC--------------------------------TATGCCAG(AGGCAAGCCCCA Rhesus
caaggcctgctatccctagaacccacgctctCAAATTCAACCTCTGAGGC--------------------------------TATGCCAG(AGGCAAGCCCCA Crab-eating macaque
caaggcctgctatccctagaacccatgctctCAAATTCAACCTCTGAGGC--------------------------------TATGCCAG(AGGCAAGCCCCA Baboon
caaggcctgctatccctagaacccacgctctCAAATTCAACCTCTGAGGC--------------------------------TATGACAG(AGGCAAGCCCCA Green monkey
CAATGCCTGATATCCCTAGAACCTATGCTCTCAAATTCAAC-TCTGAGGC--------------------------------TATGACAG(AGGCAAGCCCTG Marmoset
CAATGCCTGCTATCCCTAGAACCTATGTTCTCAAATTCAAC-TCTGAGGC--------------------------------TATGACAG(AGGCAAGCCCTG Squirrel monkey
taagttctgttgttcccagagctcatgctctGAAATGCAGTCTCTGTGTT--------------------------------TATGATAG(CAGCTACCCT-- Bushbaby
-------------ACTCATAAATAATACTCCCAAACTCAGC-TGTGTGGC--------------------------------TGAG----(--AAAAATTCT- Chinese hamster
------c-----TAGTCATAAATCATACTCCAAAACTCAGT-GTTGTGACTGAGATACAAAACAAAACACATTCTGTTTCTTTAAA----(--AAAAAGTCCA Mouse
------------------------------------------------------------------------------------------(-----------A Rat
caagtcctgctgtccccagagcccACATTTCCAACTTGAGC-CCCGTGGC--------------------------------TGCA----(--GTGAGCCT-- Guinea pig
------------------------------------------------------------------------------------------(------------ Rabbit
ccagtccg------------------gctgtgaggttcagcccctgtgac--------------------------------tacGGCCG(AGGCAAGGGCCT Pig
------------------------------------------------------------------------------------------(------------ Sheep
caatcctggccatctccaggacccatgctctCAGATTCAGCCT-----------------------------------------------(-GGCAGAGCCTG Dog
```

5'ss (human -24 to +10):

```
GCTG-AAGGAGAGA--GAGGCAG-CAG---A)GT--AAG--GGGGC  Human
GCTG-AAGGAGAGA--GAGGCAG-CAG---A)GT--AAG--GGGGC  Chimp
GCTG-AAGGGGAGA--GAGGCAG-CAG---A)GT--AAG--GGGGC  Gorilla
GCTG-AAGAAGAGA--GAGGCAG-CAG---A)GT--AAG--GGGGC  Orangutan
ACTGAAAGGAGAGA--GAGGCAG-CAG---A)GT--AAG--GGGGC  Gibbon
GCTG-AAGGAGAGA--AGGGCAG-CAG---A)GTT-AAG--GGGGC  Rhesus
GCTG-AAGGAGAGA--AGGGCAG-CAG---A)GTT-AAG--GGGGC  Crab-eating macaque
GCTG-AAGGAGAGA--GAGGCAG-CAG---A)GT---AA--GGGGC  Baboon
GCTG-AAGGAGAGA--GAGGCAG-CAG---A)GTT-AAG--GGGGC  Green monkey
GCTG-AAGGAGAGA--GAGTTAG-CAG---A)GT--AAG---GGGC  Marmoset
GCTG-AAGGAGAGA--GAGTTAG-CAG---A)GT--AAG--CGGGA  Squirrel monkey
ACTG-AAAGATAAA--GGAGGGGCCAG---A)GT--CAGGAGGGGC  Bushbaby
TCTA-AAGGTAGAA--GATATCT-GGG---G)T---GGT--TGTGT  Chinese hamster
TCTA-AAGGAAAAA--GACACCT-AGGGTAA)----GGT--TGGGC  Mouse
TCTA-AAGGAAAAA--GACACCT-GGG---A)----GGT--TGGGC  Rat
GCTG-AAGGAGGAA--GAAGCCA-CCA----)----GAG--TGGG-  Guinea pig
GCAC-AAGGACACA--GAGGCAG-CGA---G)CA--AGG--TGGGC  Rabbit
GTGC-CAGG--AGGGAGGGGCAG-CCA---G)CGTCCTG--CTG--  Pig
-------------------------------)--------------  Sheep
GCCG-AAGGAAGGA--GGAACAA-TGA---A)ATCAGGG--TGGCC  Dog
```

b

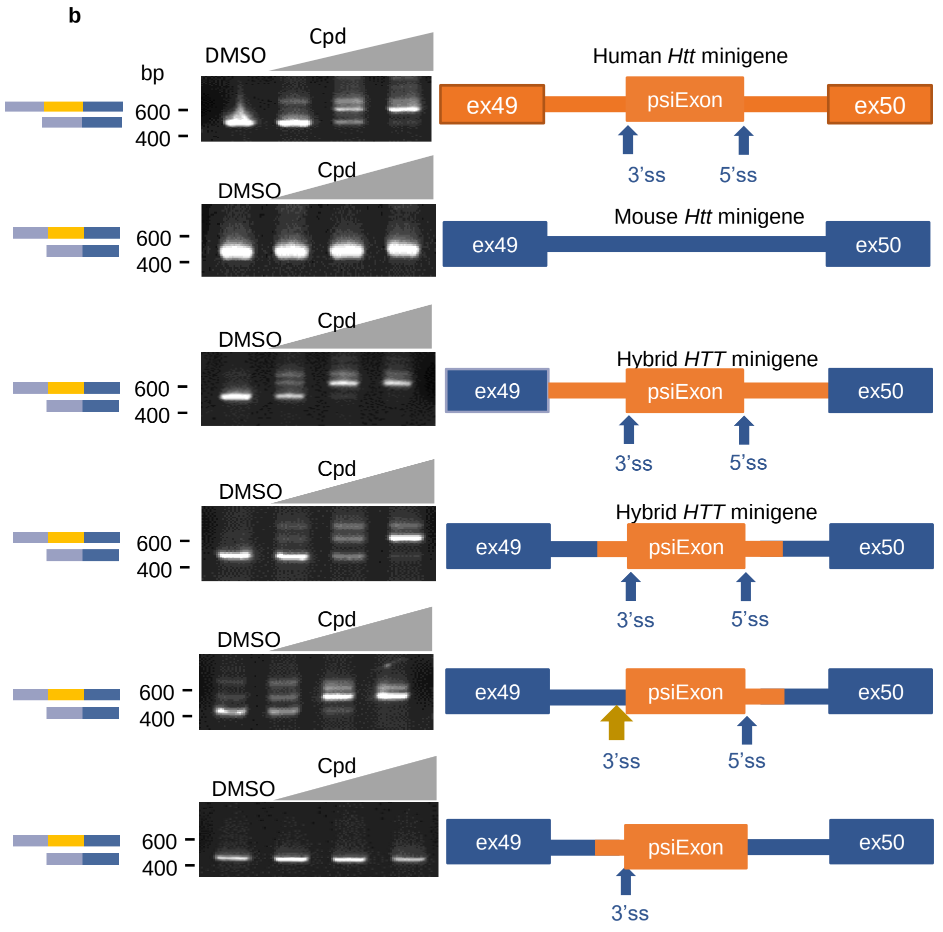

c

Putative psiExons with AGAgtaag 5'ss in human *HTT* gene in addition to stop-codon psiExon49a

intron ID	5'ss	3'ss	5'ss sequence	exon size
1	3086401	3086298	GACAGAgtaaga	104
8	3121109	3120933	ATTAGAgtaaga	177
40	3191727	3191630	TATAGAgtaaga	98
40	3193650	3193535	ACTAGAgtaaga	116

d

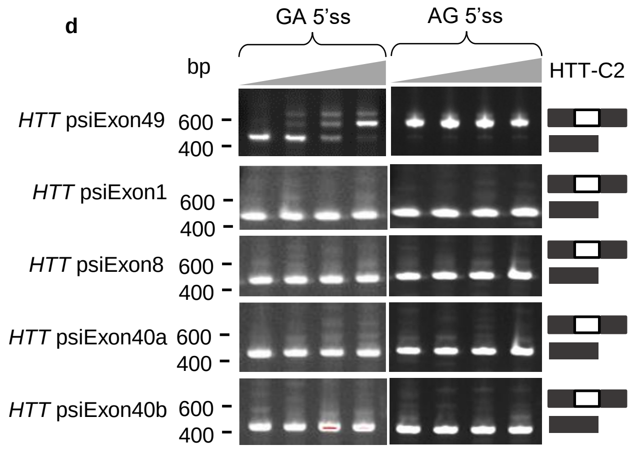

e **iExon HTT 49a: strong enhancer**

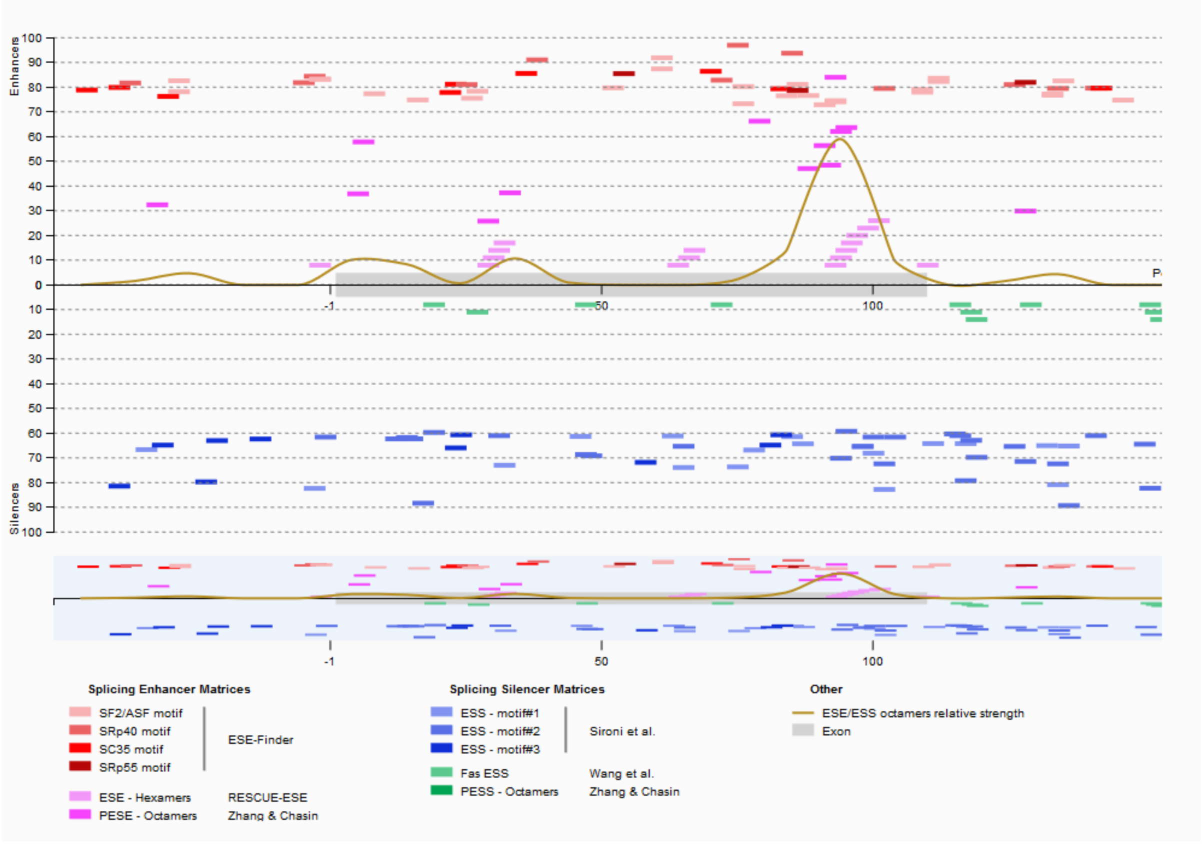

f Deletions upstream of 5'ss

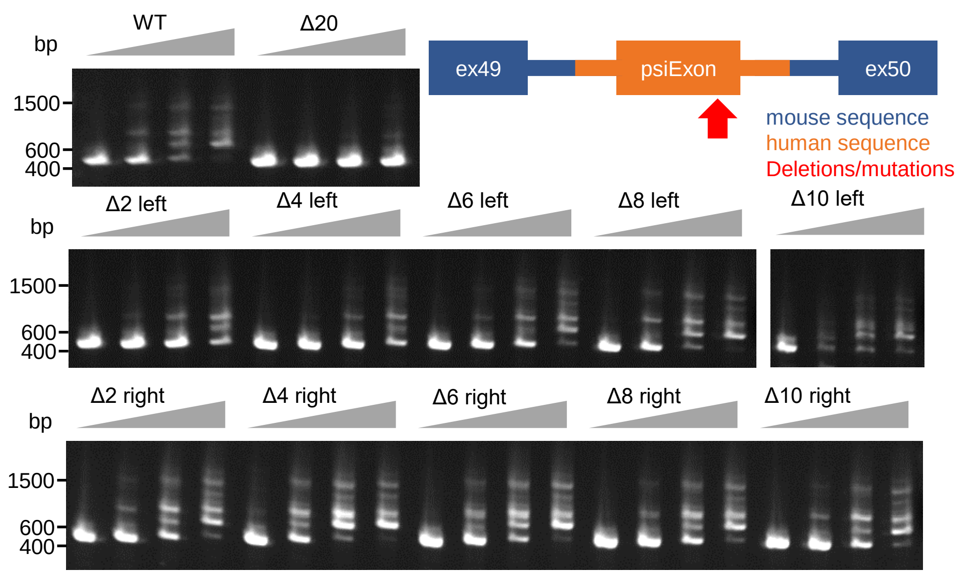

Construct	nucleotide -38 to -19
WT (control)	CAGGAGCCCACAGCGCTGAA
A = Δ20	--------------------
B = Δ2 left	--GGAGCCCACAGCGCTGAA
C = Δ4 left	----AGCCCACAGCGCTGAA
D = Δ6 left	------CCCACAGCGCTGAA
E = Δ8 left	--------CACAGCGCTGAA
F = Δ10 left	----------CAGCGCTGAA
G = Δ2 right	CAGGAGCCCACAGCGCTG--
H = Δ4 right	CAGGAGCCCACAGCGC----
I = Δ6 right	CAGGAGCCCACAGC------
J = Δ8 right	CAGGAGCCCACA--------
K = Δ10 right	CAGGAGCCCA----------

g

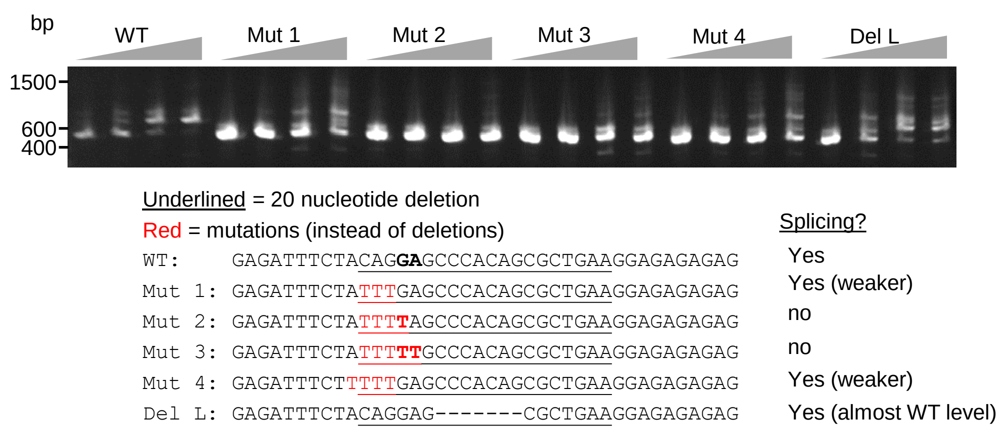

Supplementary fig. 8. Mouse and human minigene experiments conducted to characterise the upstream exonic splicing enhancers (ESE) required for compound-induced psiExon inclusion

a Multiple species sequence alignment of the human huntingtin gene *(HTT)*-psiExon49a 5' and 3' splice sites (ss). **b** Effect of various 3' and 5'ss configurations on *HTT* / mouse huntingtin gene (*Htt)* splicing in minigenes using mouse or human sequences following treatment with either dimethyl sulphoxide (DMSO) or HTT-C2. **c** Putative psiExons with AGAgtaag 5'ss in human *HTT* gene in addition to **Stop-Codon** psiExon49a. **d** Splicing assay using *HTT* intron 49 and *HTT* intron 1, 8 and 40 minigene constructs with either wild-type (wt) noncanonical (GA) or mutant canonical (AG) nucleotides at the −2, −1 positions. The data is from a single transfection experiment with multiple concentrations tested for a given construct; the "WT" control construct has been used multiple times (n>3). **e** Bioinformatic analysis of *HTT* **Stop-Codon** psiExon49a, identifying a number of potential sequences that could function as ESEs upstream of the 5'ss. **f** Effect of partial deletions upstream of the 5'ss on psiExon inclusion. The data is from a single transfection experiment with multiple concentrations tested for a given construct; the "WT" control construct has been used multiple times (n>3). **g** Effect of various mutations in the region upstream of the 5'ss on psiExon inclusion. The data is from a single transfection experiment with multiple concentrations tested for a given construct; the "WT" control construct has been used multiple times (n>3).

a

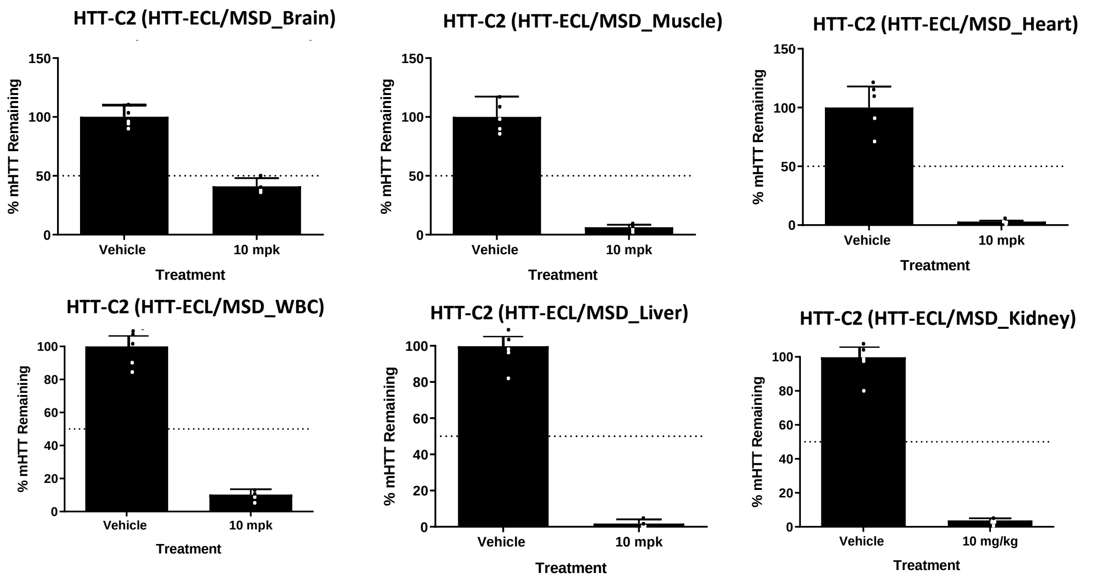

b

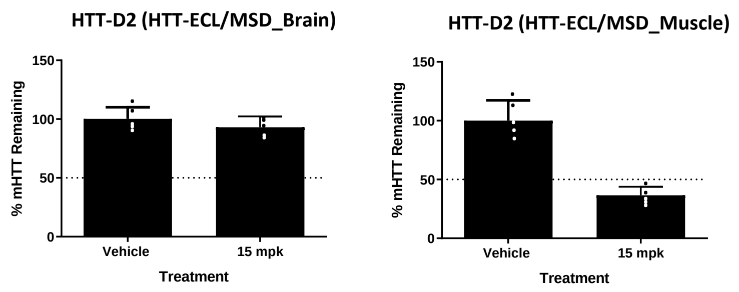

c

Compound	Structure	HTT protein lowering activity (IC_{50}), μM	Net MDR1 ER	Average $K_{p,uu}$
HTT-C1	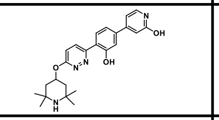	0.04	>62.7	0.084
HTT-C2	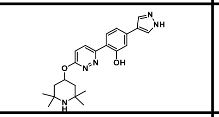	0.01	25.3	0.179
HTT-D1	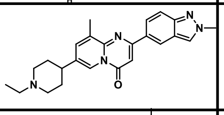	0.08	11.8	0.242
HTT-D2	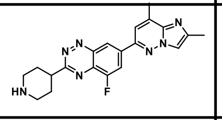	0.01	21.8	0.089
HTT-D3	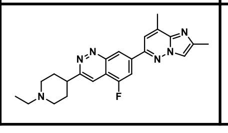	0.008	7.10	0.472

d

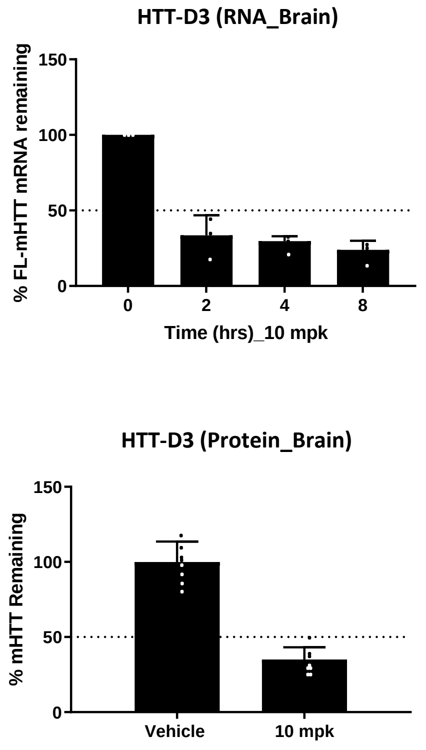

Supplementary fig. 9. HTT-C and -D series compounds and their huntingtin (HTT)-lowering effects in various mouse tissues

a Electrochemiluminescence (ECL) analysis of human HTT protein expression levels within different tissues of BACHD mice treated with 10 mg/kg HTT-C2. Graphs show percent lowering relative to vehicle control and normalised to Kirsten rat sarcoma viral oncogene homolog (KRAS), except for white blood cells (WBC). RT-qPCR and protein data represent mean ± standard deviation (SD) (error bars) of five (n=5) animals per data point. **b** ECL analysis of human HTT protein expression levels in the brains and muscles of BACHD mice treated with 15 mg/kg HTT-D2. Graphs show percent lowering relative to vehicle control and normalised to KRAS. RT-qPCR and protein data represent mean ± SD (error bars) of five (n=5) animals per data point. **c** Chemical structures, HTT protein-lowering activity, P-glycoprotein efflux (as measured by Madin-Darby canine kidney cells multidrug resistance 1 [MDCK-MDR1] permeability assay), and unbound brain partition coefficients ($K_{p,uu}$) of HTT-C and -D series compounds. **d** mHTT mRNA and protein lowering in BACHD mouse brains following treatment with HTT-D3. Graphs show percent lowering relative to vehicle control. RT-qPCR and protein data represent mean ± SD (error bars) of five animals per data point.

5'ss -2 to -1 position sequences for human Refseq-annotated transcripts

Number = 231,362

	Numbers	Fractions
AG	127562	0.55135
TG	26796	0.11582
GG	23059	0.09967
CG	14035	0.06066
AA	9824	0.04246
CT	5270	0.02278
AT	5201	0.02248
CA	5008	0.02165
GA	3113	0.01346
AC	2586	0.01118
GT	1840	0.00795
TT	1786	0.00772
CC	1751	0.00757
GC	1328	0.00574
TA	1164	0.00503
TC	1039	0.00449

44.9% (TG through GC)

Supplementary fig. 10. Canonical (AG) vs. noncanonical nucleotides in the −2, −1 positions in the 5' splice sites (ss) in human Refseq-annotated transcripts

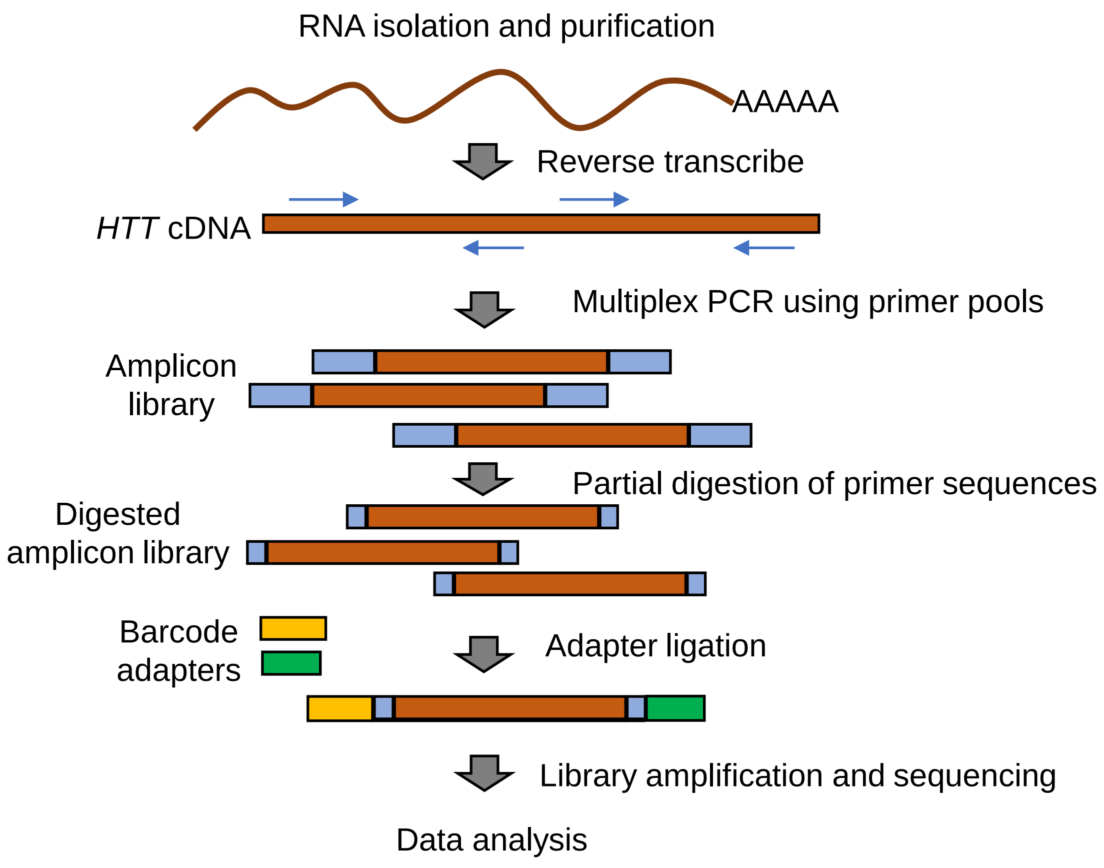

Supplementary fig. 11. AmpliSeq workflow and junction expression index (JEI) calculation

AmpliSeq workflow for polymerase chain reaction (PCR) enrichment of the huntingtin (*HTT)* exon targets.

Supplementary Tables

Supplementary table 1

RT-qPCR primers and probes used to quantify *HTT* and *GADPH* mRNA levels

Assay mix	Primer/probe	Sequence	Final concentration
HTT	Hs00918174_m1 Taqman Gene expression, 20X	-	1X
GAPDH	Forward primer	CAACGGATTTGGTCGTATTGG	100 nM
	Reverse primer	TGATGGCAACAATATCCACTTTACC	100 nM
	Probe	CGCCTGGTCACCAGGGCTGCT	75 nM

HTT, Huntingtin gene; GAPDH, glyceraldehyde 3-phosphate dehydrogenase.

Supplementary table 2

Primer sets used for primer walking assay

Forward primer	Reverse primer	*HTT* Exon Boundary	Expected Amplicon Size (bp)
CGGCTGTGGCTGAGGAG	CCAAGGTCTCCTGGACTGAT	Exon 1-Exon 6	453
CTGGATCAGCAGTGAGCATCT	TTGAAAGGACAGGGCTGCAT	Exon 7-Exon 10	498
TGACTCTGAATCGAGATCGGATGT	CAGAAGGCTGCCTGCAGT	Exon 11-Exon 14	563
GACTCTGCACCTCTTGTCCATT	CTGTTCCTCAGAGTCAGCACAT	Exon15-Exon 19	548
GGTGAGCTTTTTGGAGGCAAA	GGTCAGAATCATTGTGGCCATC	Exon 20-Exon 25	580
ACCTGCTGAAGGTGATTAACATTTGT	GGGTTGGAAGATAAGCCATCAAA	Exon 26-Exon 31	574
CAGAAAGTGTCTACCCAGTTGAAGA	AGACAGTCGCTTCCACTTGTC	Exon 32-Exon 37	640
TCCGTCCGGTAGACATGCT	AAGTCAGAATCCTCCTCTTCTCCA	Exon 38-Exon 42	709
CAGCGGCCTGTTCATCCA	CAGAAATTTCACTCATCCCTAGGCTTA	Exon 43-Exon 48	625
TGCCCAGTCATTTGCACCTT	TCTCCTCCTGCTCCATCA	Exon 49-Exon 54	756
CCAGCTGTAAGCTGCTTGGA	GTGCACCCTTCGCAGTTC	Exon 55-Exon 60	630
CACTGCCAAGCAGCTCATC	GTTGGAGAGGGACAGCATGAC	Exon 61-Exon 66	736

bp, base pair; *HTT*, Huntingtin gene.

Supplementary table 3

Gene Expression Assays (20X)

**Assay Name**	**Oligonucleotide**	**Sequence**	**Final Concentration in Reaction**
HTT assay (Thermo Fisher)	Hs00918159_m1 TaqMan Gene Expression: 60X	-	1X
GAPDH assay (Thermo Fisher)	Forward primer	CAACGGATTTGGTCGTATTGG	100 nM
	Reverse primer	TGATGGCAACAATATCCACTTTACC	100 nM
	Probe	CGCCTGGTCACCAGGGCTGCT	75 nM

Abbreviations: *GAPDH*, glyceraldehyde 3-phosphate dehydrogenase; *HTT*, Huntingtin

Supplementary table 4

Preparation of PCR Mix

**Reagent**	**Volume (µL)**	**Final Concentration**
RT-PCR buffer (2X)	5	1X
RT-PCR enzyme mixture (25X)	0.4	1X
HTT Primer/Probe (60X)	0.17	1X
GAPDH assay (20X)	0.5	1X
H_2O	1.93	-

Abbreviations: *GAPDH*, glyceraldehyde 3-phosphate dehydrogenase primer; *HTT*, Huntingtin primer; PCR, polymerase chain reaction; RT-PCR, reverse transcriptase polymerase chain reaction

Supplementary References

1. Naryshkin, N. A. et al. Motor neuron disease. SMN2 splicing modifiers improve motor function and longevity in mice with spinal muscular atrophy. *Science* **345**, 688–693 (2014).
