# Supplementary material for: Small molecule splicing modifiers with systemic HTT-lowering activity
Source: Nat Commun. 2021 Dec 15;12:7299. doi: 10.1038/s41467-021-27157-z (PMC8674292; doi:10.1038/s41467-021-27157-z)
Supplement: Supplementary file 3 — Description of Additional Supplementary Files [file 41467_2021_27157_MOESM3_ESM.pdf]

Description of Additional Supplementary Files

File Name: Supplementary Data 1
Description: **Gene expression table from RNA-Seq analysis of SH-SY5Y cells treated with 24nM and 100nM of HTT-C2 as compared to dimethyl sulphoxide (DMSO) control**. “num_”, gene read counts. “rpkm_”, the read number per kb per million total reads value representing the normalised gene expression. “L2FC_”, log 2-fold of compound-treated vs. DMSO sample. “adjSLog10P_”, -log10 based adjusted P value with a positive and negative sign representing up- and downregulation respectively. “GexType_”. Gene expression change type using fold change >1.5 and FDR <5% as cut-off (up, upregulation; dn, downregulation; nc, no significant change; na, not available [low expression in both samples]). “psiExon.coordinates”, coordinates of psiExons as identified using RNA-Seq data of SH-SY5Y cells treated with HTT-C2 or HEK293 cells treated with U1-GA variant (from Supplementary table 3).

File Name: Supplementary Data 2
Description: a Splicing analysis of RNA-Seq of HTT-C2 treatment of SH-SY5Y cells and b SMN-C3 treatment of spinal muscular atrophy (SMA) type 1 patient fibroblast. “coordinates”, the boundary of the exon or exonic region on human genome (hg19); “gene_id”, the NCBI gene ID; “refseqid”, Refseq transcript ID; “reg_len”, length (bp) of the exon or exonic region; “AS_type”, alternative splicing type (CE, cassette exon, the region starts with a 3' splice sites (ss) and ends with a 5'ss; A5SS, alternative 5'ss region; A3SS, alternative 3'ss region); “startSS_seq” and “endSS_seq”, sequences of start splice site and the end splice site positions (lowercase for intronic sequence and uppercase for exonic sequence). "exon_i_inFrame", whether the exon or exonic region (usually unannotated) is in frame? (Y, yes; N, no; NA, unavailable); "exon_i_stopCodon", whether the exon or exonic region (usually unannotated) contains at least one in-frame stop codon? (Y, yes; N, no; NA, unavailable); "startSS_supp" and "endSS_supp", start and end position annotation by transcript types (following these hierarchy: Refseq, Refseq transcript; KnG_Ens, UCSC known genes or Ensembl transcripts; None, none of above;); “PSI_SampleName”, Percent-spliced-in (PSI) of an exon or region of a sample or average value of a sample group. "deltaPSI_TestSample_ControlSample", difference of average PSI values comparing two sample groups (test vs. control); "Pfisher_TestSample_ControlSample", Fisher's Exact test P value for inclusion and skipping read counts comparing two sample groups (test vs. control); "ReguType_TestSample_ControlSample", region regulation type. (Inc, inclusion; Skp, skipping; NC, not statistically significant change; na, not available, eg. lowly expressed genes).

File Name: Supplementary Data 3
Description: **PsiExons activated by HTT-C2 or U1-GA variant**. PsiExons identified from RNA-Seq data of HTT-C2 (100nM) in SH-SY5Y cells, or U1-GA variant treatment in HEK293 cells. See the description of Supplementary table 2 for the description of the column names. We used the following criteria to select psiExons: (1) PSI increase by >20% and P value <0.001 in either dataset; (2) Event detected in both datasets: A minimal of 20 for the denominator of Percent-spliced-in (PSI) calculation was required (see Methods for details); (3) Neither the 5’ splice sites (ss) nor the 3’ss was annotated by public databases (Refseq, Ensembl or UCSC Known Genes). (4) The exon has gt-ag canonical 5’ and 3’ splice-site sequences.
