# Supplementary material for: Small molecule splicing modifiers with systemic HTT-lowering activity
Source: Nat Commun. 2021 Dec 15;12:7299. doi: 10.1038/s41467-021-27157-z (PMC8674292; doi:10.1038/s41467-021-27157-z)
Supplement: Supplementary file 7 — Reporting Summary [file 41467_2021_27157_MOESM7_ESM.pdf]

nature portfolio

Corresponding author(s): Stuart Peltz

Last updated by author(s): Oct 14, 2021

Reporting Summary

Nature Portfolio wishes to improve the reproducibility of the work that we publish. This form provides structure for consistency and transparency in reporting. For further information on Nature Portfolio policies, see our Editorial Policies and the Editorial Policy Checklist.

Statistics

For all statistical analyses, confirm that the following items are present in the figure legend, table legend, main text, or Methods section.

n/a	Confirmed	
☐	☒	The exact sample size (*n*) for each experimental group/condition, given as a discrete number and unit of measurement
☐	☒	A statement on whether measurements were taken from distinct samples or whether the same sample was measured repeatedly
☐	☒	The statistical test(s) used AND whether they are one- or two-sided<br>*Only common tests should be described solely by name; describe more complex techniques in the Methods section.*
☒	☐	A description of all covariates tested
☐	☒	A description of any assumptions or corrections, such as tests of normality and adjustment for multiple comparisons
☐	☒	A full description of the statistical parameters including central tendency (e.g. means) or other basic estimates (e.g. regression coefficient) AND variation (e.g. standard deviation) or associated estimates of uncertainty (e.g. confidence intervals)
☐	☒	For null hypothesis testing, the test statistic (e.g. *F*, *t*, *r*) with confidence intervals, effect sizes, degrees of freedom and *P* value noted<br>*Give P values as exact values whenever suitable.*
☒	☐	For Bayesian analysis, information on the choice of priors and Markov chain Monte Carlo settings
☒	☐	For hierarchical and complex designs, identification of the appropriate level for tests and full reporting of outcomes
☒	☐	Estimates of effect sizes (e.g. Cohen's *d*, Pearson's *r*), indicating how they were calculated

Our web collection on statistics for biologists contains articles on many of the points above.

Software and code

Policy information about availability of computer code

Data collection	RNAseq were performed by Novogene Co. and the fastq format files were transfered using linux command "wget" or "sftp".
Data analysis	The 5' and 3'ss MAXENT scores were calculated using MaxEntScan (http://hollywood.mit.edu/burgelab/maxent/maxentscan_scoreseq.html) representing the strength of splice sites. AmpliSeq reads (Fastq format) were mapped to human genome (hg19) using TopHat 2. RNA sequencing reads were mapped to human genome (hg19) using STAR (version 2.5). RNAseq gene expression analysis was performed using DESeq 2 (Bioconductor). PSI calculation, Fisher's exact test, k-mer analysis and statistical analysis were performed using R (3.5.1). Sequence logos were generated using Weblogo (University of California, Berkeley). All statistical tests are two-sided. The drug concentrations were acquired and processed with MassLynx 4.1 software PK parameters were estimated using the non-compartment method within Phoenix® WinNonlin® Build 8.1

For manuscripts utilizing custom algorithms or software that are central to the research but not yet described in published literature, software must be made available to editors and reviewers. We strongly encourage code deposition in a community repository (e.g. GitHub). See the Nature Portfolio guidelines for submitting code & software for further information.

Data

Policy information about availability of data

All manuscripts must include a data availability statement. This statement should provide the following information, where applicable:

- Accession codes, unique identifiers, or web links for publicly available datasets
- A description of any restrictions on data availability
- For clinical datasets or third party data, please ensure that the statement adheres to our policy

The AmpliSeq and RNA-Seq data generated in this study have been deposited in the GEO database under accession code GSE162814 (https://

www.ncbi.nlm.nih.gov/geo/query/acc.cgi?acc=GSE162814). The SMN-C3 RNA-Seq data was downloaded from the GEO database under accession code GSE62540 (https://www.ncbi.nlm.nih.gov/geo/query/acc.cgi? acc=GSE62540). Source data are provided with this paper. The code for the RNA-Seq data analysis can be found in GitHub (https://github.com/liwc01/DEDSeq). The following databases were also used: Refseq (https://www.ncbi.nlm.nih.gov/refseq/), Ensembl (https://useast.ensembl.org/index.html), and UCSC Known Genes (http://genome.ucsc.edu/).

Field-specific reporting

Please select the one below that is the best fit for your research. If you are not sure, read the appropriate sections before making your selection.

☒ Life sciences ☐ Behavioural & social sciences ☐ Ecological, evolutionary & environmental sciences

For a reference copy of the document with all sections, see nature.com/documents/nr-reporting-summary-flat.pdf

Life sciences study design

All studies must disclose on these points even when the disclosure is negative.

Sample size	No sample size calculations were performed, but several replicates were performed where appropriate for concordance and to establish consistent results.
Data exclusions	Data exclusions: no data were excluded from the analyses.
Replication	Multiple biologically independent samples were performed in order to verify reproducibility. No unsuccessful attempts at replication took place.
Randomization	No randomization took place since cell lines and mice used were genetically identical.
Blinding	No blinding took place as this was not practical and no subjective results were gathered.

Reporting for specific materials, systems and methods

We require information from authors about some types of materials, experimental systems and methods used in many studies. Here, indicate whether each material, system or method listed is relevant to your study. If you are not sure if a list item applies to your research, read the appropriate section before selecting a response.

Materials & experimental systems

n/a	Involved in the study	
☐	☒	Antibodies
☐	☒	Eukaryotic cell lines
☒	☐	Palaeontology and archaeology
☐	☒	Animals and other organisms
☒	☐	Human research participants
☒	☐	Clinical data
☒	☐	Dual use research of concern

Methods

n/a	Involved in the study	
☒	☐	ChIP-seq
☒	☐	Flow cytometry
☒	☐	MRI-based neuroimaging

Antibodies

Antibodies used	Primary antibodies: Anti-polyglutamine-expanded HTT mouse mAb (1 µg/mL; clone MW1; Developmental Studies Hybridoma Bank); anti-HTT (1:1,000; clone 1HU-4C8; Millipore; #MAB2166); anti-utrophin (UTRN) (1:250; clone DRP3/20C5 [discontinued]; Vector Laboratories; #VP-U579); antioxidoreductase-protein disulfide isomerase (PDI) (1:10,000; [discontinued] Santa Cruz; #SC20132); anti-beta actin (1:10,000; clone AC-74; Sigma; #A2228); anti-GAPDH (1:1,000; Thermo Fisher Scientific; #PAI-987); anti-alpha serine/threonine-protein kinase (AKT) (1:1,000; Cell Signalling Technology; #9272). Secondary antibodies included: Alexa Fluor 680 goat anti-mouse immunoglobulin G (lgG) (1:10,000; Thermo Fisher Scientific; #A28183), IRDye" 800 CW donkey anti-mouse lgG (1:10,000; Li-Car; #926-32212), and IRDye" 800CW donkey anti-rabbit lgG (1:10,000; Li-Car; #925-32213); anti-KRAS (1:500; Abcam; #ab137739); anti-KRAS (1:2,000; clone 2C1; LSBio; #LS-C175665-100)
Validation	All antibodies were validated by the vendors. Anti beta-actin: the antibody labels specifically beta-actin in a wide variety of tissues and species by various immunochemical techniques including immunoblotting (42 kDa) immunofluorescent staining of cultured cell lines, and IHC. Species reactivity: Drosophila, Hirudo medicinalis, carp, rabbit, wide range, pig, cat, human, rat, chicken, guinea pig, sheep, mouse, bovine, canine. Anti-AKT: Akt antibody detects endogenous levels of total Akt1, Akt2 and Akt3 proteins. The antibody does not cross-react with related kinases. Species Reactivity: human, mouse, rat, hamster, monkey, chicken, D. melanogaster, bovine, dog, pig, Guinea Pig. Anti-GAPDH: PA1-987 detects rat GAPDH. Shares 100% sequence homology with human and mouse. It has been used successfully in WB. By WB his antibody specifically detects a ~36 kDa protein representing GAPDH. Anti-HTT: species reactivity: human, rat, mouse, monkey, rabbit. Applications: ELISA, Immunocytochemistry, IHC (Paraffin), Immunoprecipitation, WB. Anti-HTT (Developmental Studies Hybridoma Bank): nonconjugated mouse monoclonal antibody to human HTT. Applications: western blot, ELISA, immunocytochemistry, immunoprecipitation, FACS. Anti-KRAS (LSBio): nonconjugated mouse monoclonal

antibody to human KRAS. Validated for IHC and WB. Anti-KRAS (Abcam): suitable for WB, IHC-P, ICC/IF; it reacts with human and predicted to work with mouse, rat, cow, zebrafish, Xenopus tropicalis.

Eukaryotic cell lines

Policy information about cell lines

Cell line source(s)	ATCC: HEK293, SHY5Y, CT26, and MDCK cells. Coriell Institute for Medical Research: GM04856/GM04857 and GM07492/ GM07491 cells. Absorption Systems: MDCK-MDR1.
Authentication	Following purchase of the cell lines none of the cell lines were further authenticated.
Mycoplasma contamination	All cell lines tested negative for mycoplasma contamination
Commonly misidentified lines (See ICLAC register)	No commonly misidentified cell lines were used in the study

Animals and other organisms

Policy information about studies involving animals; ARRIVE guidelines recommended for reporting animal research

Laboratory animals	Mice strains, BACHD is PMID: 18550760 and Hu97/18 is PMID: 23001568 , male and female mice of ages 2-4 months; mice were housed in ventilated racks with up to (no more than) 5 mice per cage; Ambient temperature was between 70 and 76 degree Fahrenheit in the animal facility; humidity was between 30 and 70%.
Wild animals	The study did not involve wild animals
Field-collected samples	The study did not involve samples collected from the field
Ethics oversight	All studies were done according to procedures reviewed and approved by the Rutgers Institutional Animal Care and Use Committee (IACUC) and University of Central Florida IACUC. All facilities have been fully accredited by the Association for Assessment and Accreditation of Laboratory Animal Care (AMLAC).

Note that full information on the approval of the study protocol must also be provided in the manuscript.
